# NOTCH3 Is a Prognostic Factor That Promotes Glioma Cell Proliferation, Migration and Invasion via Activation of CCND1 and EGFR

**DOI:** 10.1371/journal.pone.0077299

**Published:** 2013-10-15

**Authors:** Mohammad A. Y. Alqudah, Supreet Agarwal, Maha S. Al-Keilani, Zita A. Sibenaller, Timothy C. Ryken, Mahfoud Assem

**Affiliations:** 1 Pharmaceutics and Translational Therapeutics, College of Pharmacy, University of Iowa, Iowa City, Iowa, United States of America; 2 Department of Neurosurgery and Radiation Oncology, University of Iowa, Iowa City, Iowa, United States of America; 3 Department of Neurosurgery, Iowa Spine and Brain Institute, Waterloo, Iowa, United States of America; Cincinnati Children’s Hospital Medical Center, United States of America

## Abstract

Using a GWA analysis of a comprehensive glioma specimen population, we identified whole gain of chromosome 19 as one of the major chromosomal aberrations that correlates to patients’ outcomes. Our analysis of significant loci revealed for the first time NOTCH3 as one of the most significant amplification. NOTCH3 amplification is associated with worse outcome compared to tumors with non-amplified locus. NOTCH receptors (NOTCH1-4) are key positive regulators of cell-cell interactions, angiogenesis, cell adhesion and stem cell niche development which have been shown to play critical roles in several human cancers. Our objective is to determine the molecular roles of NOTCH3 in glioma pathogenesis and aggressiveness. Here we show for the first time that NOTCH3 plays a major role in glioma cell proliferation, cell migration, invasion and apoptosis. Therefore, our study uncovers the prognostic value and the oncogenic function of NOTCH3 in gliomagenesis and supports NOTCH3 as a promising target of therapy in high grade glioma. Our studies allowed the identification of a subset of population that may benefit from GSI- or anti-NOTCH3- based therapies. This may lead to the design of novel strategies to improve therapeutic outcome of patients with glioma by establishing medical and scientific basis for personalized chemotherapies.

## Introduction

Malignant glioma, the most frequent type of primary adult brain neoplasms, have dismal outcome for patients [1], [2], [3]. Complex and poorly-reproducible diagnoses, inability to accurately predict responsiveness to therapy regimens, less than optimal brain drug penetration, and genetic heterogeneity have contributed to the poor prognosis for patients with gliomas. Therefore, understanding the molecular mechanisms underlying this disease may lead to better management, to appropriate therapies and to improve the dismal outcome. Recent genomic studies in glioma have shown high levels of chromosomal instability in terms of frequent changes in DNA copy number [4], [5]. Using SNP array analyses, we previously found gain of chromosome 19 as one of these frequent chromosomal aberrations [3]. This genetic signature was observed mainly in high grade astrocytic lineages. Following copy number analysis of the most significantly deregulated genes mapped to chromosome 19, we found NOTCH3 locus (19p13.12) as one of the most significant amplification in 17% of glioma biopsies. NOTCH3 is frequently deregulated in many malignancies and its role in cancer is now firmly confirmed [6], [7], hence may play major role in gliomagenesis.

NOTCH, a highly conserved signaling pathway among several species, plays an important role in cell fate decisions during development, maintenance of stem cell proliferation and vascular formation [8]. NOTCH proteins are type I transmembrane receptors with a central role in the pathogenesis of human cancer [9]. NOTCH signaling is mediated by cell-to-cell contact and initiated via binding by ligands triggering two successive cleavages by the metalloproteinase tumor necrosis factor-α-converting enzyme (TACE) and the presenilin–γ-secretase complex leading to the release of the active intracellular domain (ICD). The translocation of the ICD into the nucleus leads to its binding to the CSL repressor complex (CBF1/RBP-Jk, Suppressor of Hairless, LAG-1) and recruitment of the co-activator protein mastermind-like 1 (MAML1). This transcriptional complex is required for activation of NOTCH target genes such as HES and HEY families, cyclin D1 (CCND1) and c-MYC [10], [11] that ultimately results in activation of cell cycle and inhibition of apoptosis. Interestingly, NOTCH pathway also regulates development of stem cells and activates the epithelial-to-mesenchymal transition and renders some glioma more invasive and aggressive [12], [13]. Stem cell niche and brain cancer stem cells roles in glioma are increasingly investigated and constitute a potential target in anticancer research. Moreover, NOTCH modulators, such as γ-secretase inhibitors, have been shown to influence the proliferation and differentiation of the stem cells niche [14], [15].

There is increasing evidence supporting the association of one or more NOTCH pathways in a wide range of neoplasms including development of malignant glioma. For instance, siRNA targeting NOTCH1 inhibits proliferation and invasion of glioma cells and induces apoptosis *in vitro* and *in vivo* [16]. Moreover, constitutive expression of NOTCH2 into neural stem cells (NSCs) results in their transformation into malignant tumor stem cells in transgenic mice [17]. However, NOTCH3, which is expressed at much higher levels in brain compared to other NOTCHs, has not yet been implicated in gliomagenesis. Some studies have shown a redundancy among NOTCH genes and that some of them may act also as tumor suppressors in specific contexts and tissues. However, their significance is unknown.

In the current study, we found that NOTCH3 was significantly overexpressed in a subset of high grade gliomas at both the mRNA and protein levels. We investigated the functional role of NOTCH3 in malignant glioma cell lines to further elucidate the significance of NOTCH3 gene amplification. In addition, the cellular and molecular effects of NOTCH3 knockdown were assessed, therefore providing us major insights into the role of NOTCH3 in gliomagenesis.

## Materials and Methods

### Genomic Data

Sixty tumor biopsies from patients with glioma were harvested at the University of Iowa Department of Neurosurgery and analyzed using a genome-wide analysis exploration as previously described [3]. Participants provided their written informed consent. The study was approved by the University of Iowa institutional review board (HawkIRB; IRB#200707727).

### Cell Culture, Lentivirus Preparation and Transduction

U87-MG human glioma cell line was obtained from ATCC (Manassas, VA) and U251-MG human glioma cell line was a kind gift from Dr. Maltese [18]. pLKO.1 lentiviral vectors encoding short hairpin RNA (shRNA) targeted to human NOTCH3 or scrambled shRNA (Scr) as control, were purchased from ThermoScientific-OpenBiosystems (Huntsville, AL). The sequence of the selected shRNA is sh235: ATCTCCAGCATTACTACCGAG. For lentivirus production, TSA201 cells were cultured in six-well plates. Cells were transfected with shRNA plasmids along with envelope and packaging plasmids (VSV-G and PAX2) using Polyfect transfection reagent (Qiagen, Germany) according to the manufacturer’s protocol. Subsequently, lentivirus supernatants were harvested at 24 and 48 hours and pooled. For transduction, lentivirus supernatants were added to U87-MG and U251-MG glioma cells with 8 µg/ml polybrene (Sigma-Aldrich, St. Louis, MO). 1 µg/ml puromycin was used to select the positive clones 48 hours post transduction (Sigma-Aldrich, St. Louis, MO). 72 hours after puromycin selection, cells were used for cellular and molecular assays.

### RNA Isolation, Reverse Transcription and Real Time PCR

Total RNA was extracted from cultured cells using TRIzol reagent according to the manufacturer’s protocol (Life technologies, Carlsbad, CA). RNA quality was assessed on a 1% agarose gel and quantified at 260/280 nm. 5 µg RNA was used for reverse transcription using Superscript III/OligodT according to the standard protocol (Life technologies, Carlsbad, CA). 50 ng of cDNA was then used for further analyses. Quantitative real time PCR analysis was performed using specific primers for NOTCH1 (forward primer, CCTTTGTGCTTCTGTTCTTCG and reverse primer, CTCATTCTGGTTGTCGTCCAT), NOTCH2 (forward primer, ATGGTGGCAGAACTGATCAAC and reverse primer, TTGGCAAAATGGTCTAACAGG), NOTCH3 (forward primer, AGGTGATCGGCTCGGTAGTAA and reverse primer, TTACTACCGAGCCGATCACCT), NOTCH4 (forward primer, GATCCTCATGTGTGTGTGACG and reverse primer, GTGGGTCCTGTGTAGCCTGTA), HEY2 (forward primer CGTCGGGATCGGATAAATAA and reverse primer, GCACTCTCGGAATCCTATGC), C-MYC (forward primer, CCTACCCTCTCAACGACAGC and reverse primer, CTCTGACCTTTTGCCAGGAG), CCND1 (forward primer, GTTCATGGCCAGCGGGAAGAC and reverse primer, CTGCCGTCCATGCGGAAGATC) and GAPDH as an internal control (forward primer, ACCACAGTCCATGCCATCAC and reverse primer, TCCACCACCCTGTTGCTGTA).

### Western Blot

NOTCH3 protein expression was analyzed using NOTCH3 primary antibody recognizing an epitope on the intracellular domain (ab23426, Abcam, Cambridge, MA). GAPDH primary antibody (ab8245, Abcam, Cambridge, MA) was used as a loading control. Immunoreactive proteins were detected using horseradish peroxidase-conjugated secondary antibodies and enhanced chemiluminescence (ECL) according to the manufacturer’s protocol (GE Healthcare, Piscataway, NJ).

### MTT and Growth Curve Assays

Cell viability was assessed using the MTT colorimetric assay. 5000 cells/well were plated in a 96 well plate in triplicate for each condition and allowed to grow for 96 h. Cell viability was assessed by adding 10 µl of filter sterilized 3-(4, 5-dimethylthiazol-2-yl)-2,5-diphenyltetrazolium bromide (MTT) solution at 0.5 mg/ml to each well. Following 4-hour incubation the media was removed and the blue formazan crystals trapped in cells were dissolved in DMSO. Absorbance was measured at 570/690 nm using a microplate spectrophotometer (Spectra Max Plus, Molecular Devices, CA). The results are expressed as a percentage of viable cells relative to mock cells.

For growth curve assay, 15,000 cells/well were seeded in 6-well plates in triplicates and allowed to grow for 1, 5 and 10 days for U87-MG cells and 1, 3 and 5 days for U251-MG cells. At each time point, the wells were rinsed twice with PBS, fixed for 5 minutes in ethanol and stained with 0.1% crystal violet for 10 minutes. Stained cells were washed twice with ddH_2_O and allowed to air-dry. Methanol was then added to each well to dissolve the stain. Absorbance was measured at 540 nm and results were expressed as the average growth (A540 nm of methanol-solubilized cell stain at defined time points) ± SE.

### Soft Agar Colony Forming Assay

Monitoring of anchorage-independent growth of glioma cells was performed using a soft agar colony formation assay. Cells (15000 cells/well) were plated, in a six-well plates, in 0.35% agar in DMEM medium with 20% FBS over a solid layer of 0.55% agar. Plates were then incubated for 3–4 weeks at 37°C. The resulting colonies were stained with 0.1% crystal violet and visualized by light microscopy. Colonies were counted in 10 random fields at 4× magnification. Results were expressed as average colony count ± SE from three independent experiments.

### Flow Cytometry Analysis of the Cell Cycle

U87-MG cells were synchronized using serum starvation for 24 hours then transiently transduced with NOTCH3 sh235 or scrambled shRNA lentiviruses. After gene knockdown, cell cycle analysis was performed using flow cytometry with propidium iodide (PI). Briefly, cells were harvested with 0.05% trypsin and the pellets (5×10^5^ cells) were suspended in 500 µl cold, hypotonic PI staining solution (50 µg/mL propidium iodide, Sigma Aldrich, St. Louis, MO) containing 0.1% Triton X-100 in 0.1% sodium citrate solution and RNase A (10 µg/mL). The stained cells were incubated for 15 minutes on ice. The cell cycle analysis of nuclei suspension was analyzed using flow cytometry (Becton Dickinson LSR II, Franklin Lakes, NJ). ModFitTM software was used to analyze the resulted DNA content curves and generate DNA content histograms.

### DNA Extraction and Analysis

Cellular apoptosis was analyzed with DNA laddering. Briefly, U87-MG cells were transiently transduced with NOTCH3 sh235 or scrambled shRNA lentiviruses. After gene knockdown, media was changed into fresh DMEM with 10% FBS. After 48 hours, DNA was extracted from the cells using a standard technique (DNAeasy, Qiagen, Germany). DNA laddering was analyzed on a 1% agarose gel electrophoresis using 2 µg of total DNA. Intact genomic DNA was shown as a unique band at the top of the gel, while fragmented DNA populations were represented by a ladder pattern corresponding to the internucleosomal processing.

### Wound Healing and Transwell Assays

Cell migration was measured using the scratch wound healing assay as described previously [19]. Briefly, after transduction, U87-MG cells were cultured in 6-well plate until confluence and then serum starved for 6 hours. A wound was introduced in the confluent monolayer of glioma cells using a pipette tip, and maintained under serum free media until closure of the wounds. The wounds’ pictures were analyzed using NIH-Image J software. Cell migrations at 12 and 24 hours were expressed as percentages of the baseline wound area. The results represent the average of cell migration rate ± SE.

For invasion assay, BD BioCoat Matrigel invasion chambers (BD Biosciences, Bedford, MA) were used according to the manufacturers’ instructions. The transwell membrane filter inserts with 8-µm pore size were rehydrated and placed in a 24-well tissue culture plates. U87-MG cells were transiently transduced with NOTCH3 sh235 or scrambled shRNA lentiviruses. After trypsinization, cells were counted, centrifuged and resuspended into serum-free medium. 50 K cells/well from serum-free cell suspension were added to the top chambers, and 10% serum-containing media was added to bottom chambers then the plates were incubated at 37°C. 48 hours later, the invading cells were fixed with cold ethanol for 5 minutes and stained with 0.1% crystal violet solution in 20% ethanol for 30 minutes. The non-invading cells were removed from the upper surface of the membrane using a cotton swab. Stained cells were washed twice with PBS, left to air-dry and counted in four random fields per sample using NIH-image J software. Results were expressed as average values ± SE relative to the mock invading cells.

### Statistical and Survival Analyses

Statistical significance was detected using one-way ANOVA. Multiple group comparisons were analyzed with Tukey’s multiple comparison tests. Kaplan-Meier analysis was used to generate the overall survival curve and the log rank test was used to compare median survival. Statistical analyses were performed using SAS 9.3 and Partek (Partek, St. Louis, MO). P<0.05 was considered to be statistically significant. All data are represented as mean value ± SE of at least a triplicate experiment.

## Results

### NOTCH3 is a Candidate Oncogene in Malignant Glioma

**Figure 1:**
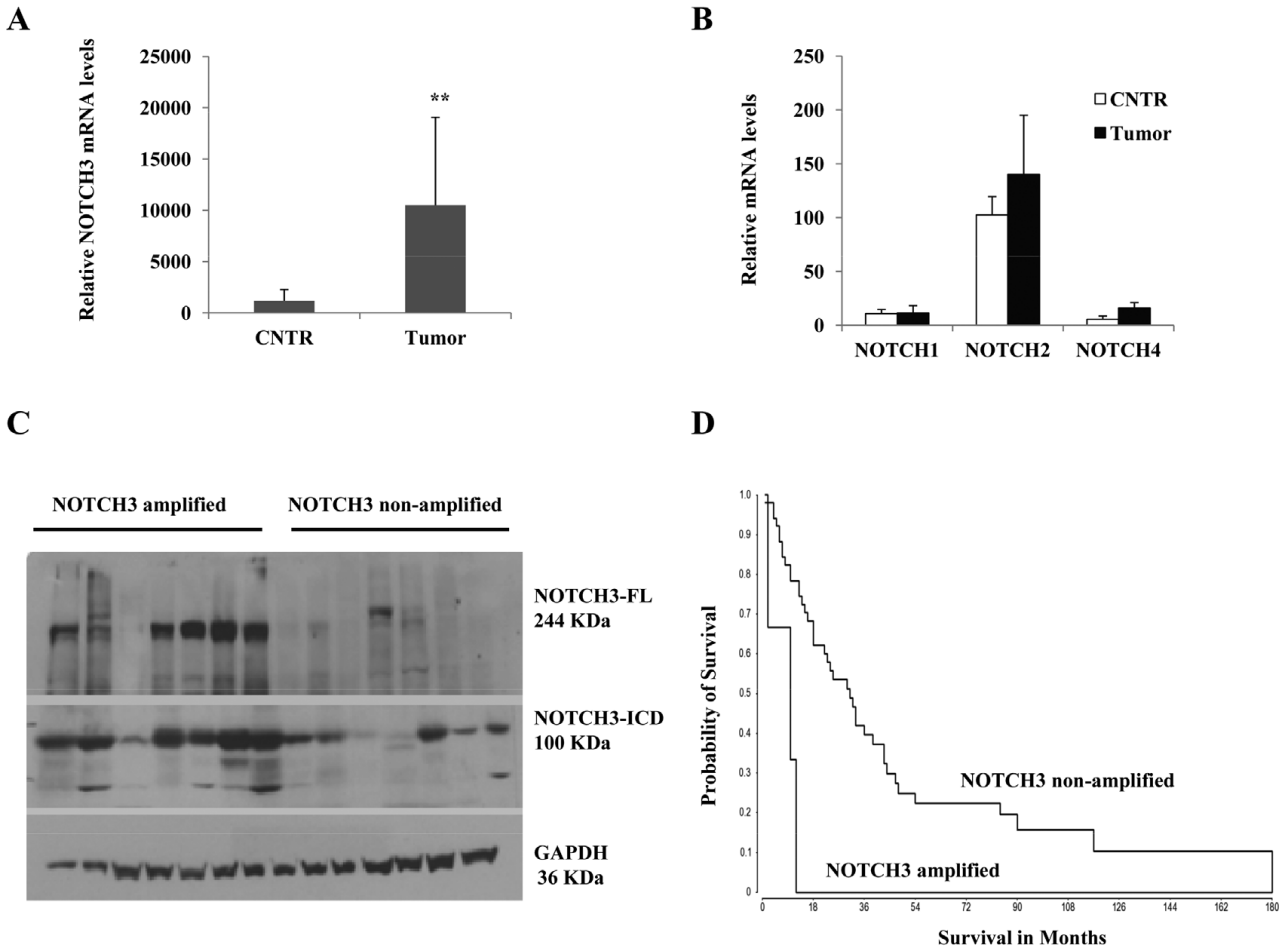
NOTCH3 alterations analysis in malignant glioma samples. (**A**) NOTCH3 gene expression in representative specimens using quantitative-real time PCR showing high levels of NOTCH3 transcripts in various tumor specimens compared to non-tumor samples (epilepsy biopsies; CNTR). (**B**) Relative mRNA levels of other NOTCH members in tumor specimens compared to non-tumor biopsies. (**C**) NOTCH3 protein expression comparisons in representative tumor specimens using western blot showing different protein levels according to NOTCH3 locus status. Tumors with amplified NOTCH3 locus showing high level of full length NOTCH3 protein (∼244 KDa) and its cleaved intracellular domain (NICD3 ∼100 KDa). GAPDH was used as a loading control. (**D**) Kaplan-Meier estimate of probability of survival stratified according to NOTCH3 genomic status (Log Rank test, P = 0.00098; median survival 10 vs. 28 months). **indicates p<0.01 (ANOVA).

We previously identified a novel chromosome 19 gain genetic signature in high grade gliomas that associates with poor outcome [3]. To narrow down the candidate genes lists that may be responsible for this grim outcome, we selected top significant genes mapped to chromosome 19 for further statistical association with prognosis, patients’ outcome and response to therapies. These genes contribute to many important pathways, such as MAPK, JAK-STAT, ubiquitination, NOTCH and VEGF pathways. The analysis revealed NOTCH3 locus (19p13.12) as the most significant gene amplification (p = 1.4×10^−8^, Anova). Therefore, we selected NOTCH3 for further expression analysis. Using quantitative real time PCR, we assessed NOTCH3 transcript levels in glioma biopsies and found that NOTCH3 RNA levels were significantly higher in tumor vs. non tumor specimens (Fig. 1A). To show that NOTCH3 is the major element in this pathogenesis, other NOTCH members were also analyzed at the transcript levels. Our results showed no significant difference in NOTCH1, 2 and 4 mRNA content between tumor and non tumor specimen (Fig. 1B). Moreover, these genes are expressed at much lower levels compared to NOTCH3. Indeed, mRNA levels of NOTCH3 are 3–5 time higher than NOTCH2, 5–10 time higher than NOTCH1, and 10–20 time higher than NOTCH4 (data not shown). In addition, we used immunoblot to compare NOTCH3 protein expression in glioma patient biopsies clustered according to chromosome 19 status. As expected, NOTCH3 protein levels were significantly higher in NOTCH3 locus amplified biopsies than their non-amplified locus glioma counterparts. This protein overexpression was observed at both full length NOTCH3 and its cleaved intracellular domain (NICD3) (Fig. 1C). Furthermore, we evaluated the prognostic significance of NOTCH3 amplification using Kaplan-Meier survival analysis and found out that NOTCH3 genomic status was associated with patient outcome (P = 0.00098, median survival 10 vs. 28 months, Log-Rank test) (Fig. 1D). The data indicate that patients with amplified NOTCH3 (17%) have poorer outcomes compared to patients with non amplified locus. NOTCH3 status at both gene and transcriptomic levels was also independently shown to predict outcome in NCI Rembrandt dataset (not shown). Together, these data strongly provide evidence that NOTCH3 could be a critical element in gliomagenesis.

### NOTCH3 Modulates Glioma Cell Proliferation

**Figure 2:**
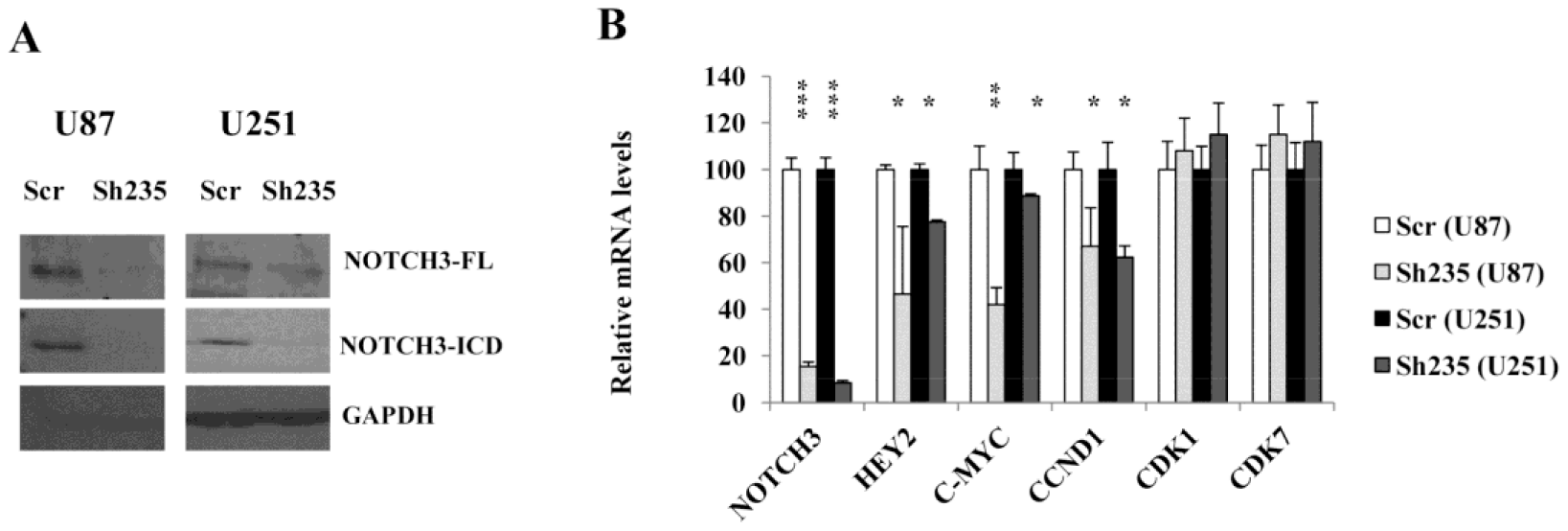
NOTCH3 knockdown in U87-MG and U251-MG cells reduced expression of NOTCH3 and its target. (**A**) Western blot of U87-MG and U251-MG cells showing silencing of NOTCH3 protein expression at both full length and cleaved forms following shRNA treatment. GAPDH was used as loading control. (**B**) Quantitative-real time PCR showing downregulation of NOTCH3 transcripts and several of its known targets in U87-MG and U251-MG cells following shRNA knockdown. *indicates p<0.05, **indicates p<0.01 and **indicates p<0.001 (ANOVA and Tukey’s multiple comparison test).

To determine the functional role of NOTCH3 in malignant glioma, we first produced NOTCH3 specific shRNA lentivirus to establish an *in vitro* model for further experiments. We screened several ShRNAs and selected sh235 that showed maximum NOTCH3 knockdown (over 80%) without altering the levels of other NOTCHs. Efficiency of gene knockdown using sh235 was verified on the 2 malignant glioma cell lines U87-MG and U251-MG using immunoblot and real time PCR (Figs. 2A and 2B). Downregulation of NOTCH3 protein levels at both full length (FL) and cleaved form (NICD3) with sh235 was consistent with the corresponding decrease in NOTCH3 mRNA levels. Indeed, Sh235 reduced NOTCH3 mRNA levels by more than 80%. In order to confirm that sh235 effectively blocked NOTCH signaling, we evaluated the gene expression levels of the known downstream targets of NOTCH; HEY2, C-MYC and CCND1. Their expression was remarkably reduced with sh235 (Fig. 2B) further confirming the knockdown efficacy. To rule out that these reductions may result from cell death, CDK1 and CDK7 mRNA levels were analyzed and showed that their expression is sustained confirming thus that only positive NOTCH3 targets are altered. Consistent with U87-MG, sh235 was also efficient in silencing NOTCH3 in U251-MG cells at both NOTCH3 mRNA and protein levels (Figs. 2A and 2B).

**Figure 3:**
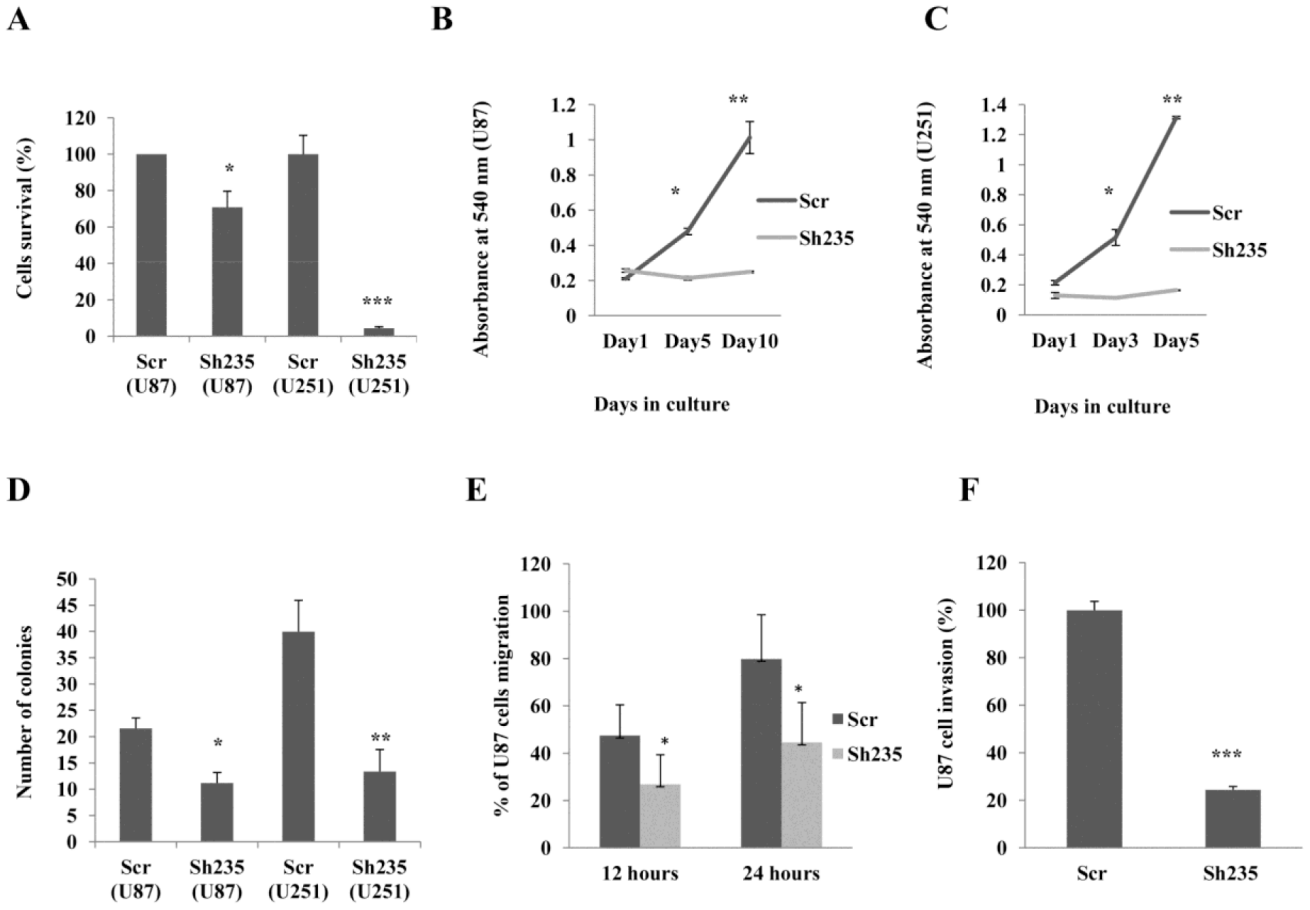
NOTCH3 knockdown in U87-MG cells represses glioma cells growth and hinders cell migration and invasion. (**A**) Cell viabilities of U87-MG and U251-MG cells following NOTCH3 knockdown were determined using MTT assay. The data represent mean ± SE of three independent experiments performed in triplicate. (**B**) Growth curves U87-MG and (**C**) U251-MG cells resulting from specific NOTCH3 knockdown were monitored with crystal violet staining at indicated time points. The results are plotted as the average growth (A_540nm_) ± SE of three independent experiments. (**D**) Colony formation ability of glioma cells was determined with soft agar colony formation assay. Data represent mean ± SE of three independent experiments. (**E**) Wound healing assay for U87-MG cells. ImageJ software was used to measure changes in cell migration per time in U87-MG cells transduced with sh235 (N = 20) or control (N = 23) lentivirus. Results are expressed as relative migration to the baseline. (**F**) Matrigel cell invasion assay. U87-MG cells expressing sh235 or scrambled control lentivirus were seeded into the upper chamber of the matrigel coated transwells. Invasive cells were stained with crystal violet, photographed under an inverted light microscope and quantified using ImageJ software in four representative fields. *indicates p<0.05, **indicates p<0.01 and **indicates p<0.001 (ANOVA and Tukey’s multiple comparison test).

The biological effect of NOTCH3 knockdown on cell viability was measured by MTT cell proliferation assay. Interestingly, the viability of U87-MG and U251-MG cells was reduced after NOTCH3 knockdown compared to the mock. U87-MG and U251-MG cells viability was significantly decreased by 40% and 80% respectively compared to the scrambled mock control (Fig. 3A). We further confirmed these findings by studying the growth curves of transduced U87-MG and U251-MG cells at different time points. NOTCH3 knockdown significantly reduced the growth of U87-MG cells by 60 and 80% on day 5 and day 10 respectively (Fig. 3B). U251-MG cell growth was reduced by more than 70% on day 3 and day 5 (Fig. 3C). The growth inhibitory effect of NOTCH3 knockdown was maintained for 5–10 days of observation, whereas mock cells were not affected. Moreover, we examined whether NOTCH3 knockdown alters anchorage-independent cell proliferation using soft agar colony formation assay. We observed that loss of NOTCH3 significantly suppressed colony formation of glioma cells on soft agar (Figs. S1 and 3D). There was an approximately 2–3 fold reduction in the number of colonies when U87-MG and U251-MG cell lines were transduced with NOTCH3 shRNA lentivirus. Taken together, these data suggest a major role for NOTCH3 in malignant glioma cell proliferation.

### NOTCH3 Promotes Glioma Cell Migration and Invasion

Based on previous results, we hypothesized that NOTCH3 may promote glioma cell migration and invasion. These two properties are hallmark of malignant glioma. Using an *in vitro* wound healing assay, specific NOTCH3 knockdown resulted in marked suppression of U87-MG cell migration at 12 and 24 hours (Fig. S2A). Quantitative analysis showed significant reduction of cell migration by 50% (Fig. 3E). In parallel, we confirmed these findings by studying the effects of NOTCH3 knockdown on cell invasion using matrigel invasion chambers that simulate the major components of extracellular matrix (ECM) and mimic the *in vivo* process of cell invasiveness. The results of the invasion assay indicate that NOTCH3 knockdown had a prominent inhibitory effect on U87-MG cell invasion (Figs. 3F and S2B). Sh235 significantly suppressed cell invasion by more than 75%. Thus, for the first time our studies show that NOTCH3 signaling promotes both migratory and invasive abilities of glioma cells and that its function is not limited only to stem cells development and proliferation.

### Loss of NOTCH3 Alters Cell Cycle and Induces Apoptosis in U87-MG

**Figure 4:**
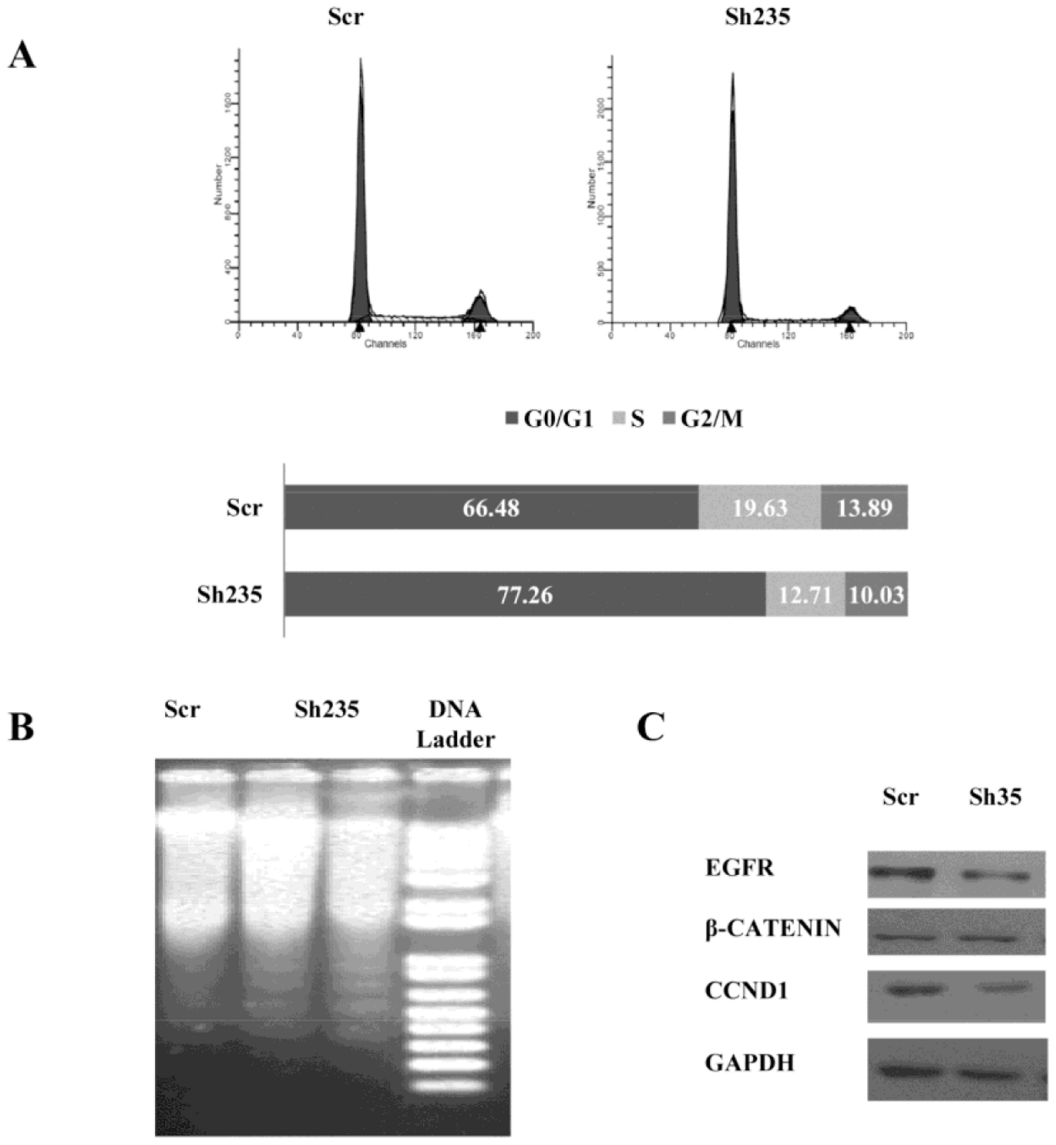
NOTCH3 knockdown alters cell cycle progression and induces apoptosis via downregulation of Cyclin D1 and EGFR. (**A**) Cell cycle profile of U87-MG cells on day 5 post-transduction with shRNA or Mock virus. Cells were synchronized to the G1/S boundary with serum free media and treated with sh235. Cell cycle was assessed by propidium iodide staining followed by FACS analysis. Results are shown in histogram distribution of the 3 major cell cycle steps. (**B**) NOTCH3 knockdown induced apoptosic DNA fragmentation in U87-MG cells. Extracted DNA was analyzed by DNA integrity on agarose gel. Visible DNA ladder in U87-MG cells following NOTCH3 knockdown is shown. (**C**) Immunoblot analysis of potential NOTCH3 targets protein content following NOTCH3 knockdown.

Since NOTCH3 knockdown resulted in growth suppression of glioma cells, we further investigated the underlying molecular mechanism related to this growth inhibition by analyzing cell cycle and apoptosis following inhibition of NOTCH3 activity. We performed flow cytometry analysis of cell cycle in mock and NOTCH3 shRNA- transduced U87-MG cells. Cell cycle kinetics showed that NOTCH3 knockdown significantly increased the percentage of U87-MG cells at G0/G1 phase (Fig. 4A). This result is expected as sh235 downregulated CNND1 (Fig. 2B) that has a crucial role in cell cycle progression in G1 phase [20]. We then explored whether NOTCH3 knockdown-mediated cell cycle arrest could have an effect on U87-MG cellular apoptosis. NOTCH3 knockdown clearly induced cellular DNA fragmentation (Fig. 4B) in correlation with the cell cycle analysis data. Our findings therefore indicated that NOTCH3 knockdown caused cell growth suppression at least by down-regulating CNND1 and EGFR expression (Fig. 4C) and inducing cell cycle arrest with subsequent apoptosis. Based on cell cycle data and on the intensity of DNA apoptotic fragmentation bands, we estimated that approximately 10–15% of cells were in apoptosis.

To further elucidate the role of NOTCH3 signaling in glioma, RNA and protein levels of potential NOTCH3 targets were assessed using real time PCR and immunoblot. As shown in figures 2B and 4C, knockdown of NOTCH3 is associated with significant reduction in levels of Cyclin D1 (CCND1) and EGFR proteins and transcripts. In relation to role of NOTCH3 in cell migration, we also analyzed β-Catenin levels because it is a major regulator of cell migration and diffusion in surrounding tissues. As shown in Fig. 4C, β-Catenin levels were however not altered. Phosphorylation status of β-Catenin was not analyzed as NOTCH pathway doesn’t directly alter phosphorylation status of several known targets. Therefore migration and diffusion of glioma cells via β-Catenin may not be altered in a NOTCH dependent manner in our conditions.

## Discussion

Gene amplification is a well-known ubiquitous mechanism of oncogene activation in human cancers [21]. In our previous genomic study of glioma tumors, we identified several chromosomal amplifications that associate with patients’ outcomes. Specifically, gain of multiple copies of chromosome 19 is one of the major chromosomal instabilities frequently associated with short term survival [3]. We showed that gains of chromosome 19 are associated with the aggressive clinical behavior of gliomas which is mainly related to resistance to therapy, tumor hyper-vascularization and to short term survival [3], [22], [23]. In our glioma population, detailed genomic analysis of chromosome 19 amplifications revealed NOTCH3 as one of the most significant amplification. The data indicate that patients with amplified NOTCH3 have poorer outcomes compared to those with normal NOTCH3 locus. Using quantitative real time PCR, RT-PCR and immunobloting, we further analyzed the level of NOTCH3 expression and confirmed that NOTCH3 was amplified in several glioma biopsies.

There are four NOTCH receptors (NOTCH1-4) in mammals that have a specific pattern of expression. Therefore we decided to explore their expression levels to rule out involvement of other NOTCHs. This analysis revealed no major changes in NOTCH1, NOTCH2 and NOTCH4 transcript levels in our glioma population. To further specifically implicate NOTCH3 in cancerogenesis, down regulation of NOTCH1 and NOTCH2 using siRNA were found to have no effect on proliferation, whereas knockdown of NOTCH3 significantly suppressed proliferation and promoted apoptosis in breast cancer cells [24]. Moreover, NOTCH3 amplifications in other cancers such as non-small cell lung cancer [15], ovarian cancer [6] and T-cell leukemia [25] have also been found. However, role of NOTCH3 has not been studied in gliomagenesis despite the fact that it plays central role in brain development and in cancer stem cell niche maintenance [26],[27]. Only 2 recent studies by Gaiano and Eberhart’s groups have implicated NOTCH3 in brain development and brain tumors [26], [28] by demonstrating that retroviral injection of the active NICD3 into forebrain ventricles of mouse embryos resulted in formation of postnatal choroid plexus tumors.

Our finding of high frequency NOTCH3 gain in glioma without significant alteration of other NOTCH members is novel and suggests that these alterations may be early and major events in gliomagenesis. We suspect that NOTCH3 is a critical element in glioma based on the frequency of gain of chromosome 19 and amplification of NOTCH3 locus (19p13.12) and its positive association with worst patients’ outcomes. These observations add another layer proving the potential role of NOTCH3 in cancer.

In the present study, we explored the oncogenic role of NOTCH3 in malignant glioma by downregulating NOTCH3 expression in glioma cell lines using shRNA. Our results elucidate for the first time the pivotal role of NOTCH3 knockdown in suppression of glioma cell proliferation and invasion, arrest of cell cycle and induction of apoptosis. Our data are consistent with previous studies about the oncogenic role of NOTCH3 in other malignancies [6], [29], [30]. Inhibition of NOTCH3 resulted in significant decrease of glioma cell survival and this effect of growth suppression was maintained to the extent that we were not able to create stable cell lines that express NOTCH3 shRNA since the cells died 2–3 weeks following NOTCH3 knockdown. This was further confirmed by the inability to obtain xenografts in NOD/SCID mice as mock cells generated massive tumors in a short period of time (data not shown). These results suggest that malignant glioma cells are dependent on NOTCH3 expression and their survival is abolished after NOTCH3 inactivation, demonstrating an oncogene addiction phenomenon [31].

The role of NOTCH3 in glioma invasion, adhesion and diffusion was not previously known. Previous studies have shown that NOTCH1 signaling could promote cellular adhesion capabilities to the ECM via activation of natively expressed fibronectin receptor and α5β1-integrin [32]. Although NOTCH receptors may have different roles in certain contexts, the redundant effect of NOTCH receptors is well established [26], [33]. In particular, we report anti-glioma effect of NOTCH3 knockdown similar to that previously reported about silencing of NOTCH1 [16]. These data strongly support the role of NOTCH pathway in cell-ECM adhesion besides the well-established cell-cell interaction. The anti-proliferative effects of NOTCH3 knockdown in glioma cells extended to *in vitro* assays of tumorigenicity, in which glioma cells lacking NOTCH3, were not able to proliferate independently of external signals, as confirmed in soft agar colony formation. Collectively, our findings suggest that NOTCH3 signaling is crucial for the proliferation of glioma cells which is consistent with previous studies in other cancers such as lung and breast cancer [24], [34].

We revealed that NOTCH3 knockdown reduces the expression level of CCND1 and c-MYC which are known targets of NOTCH and key regulators of cell cycle and proliferation. CCND1 downregulation is well characterized to decrease activation of cyclin-dependent kinases Cdk4 and Cdk6, that are not only required for G1 to S phase transition during cell cycle progression but also are major players in interaction with stromal cells that sustain tumor growth [35]. Downregulation of CCND1 leads to accumulation of a subset of glioma cells at G0/G1 which might be the underlying mechanism of low proliferative capabilities of glioma cells upon silencing of NOTCH3 [20]. C-MYC is essential for glioma cell cycle progression and proliferation and loss of c-MYC abrogates their tumorgenic potential [36]. Our data are in agreement with the central role of c-MYC in regulating glioma cancer stem cell proliferation. These data suggest that NOTCH3 has a pivotal role in regulation of glioma cell proliferation via at least c-MYC and CCND1.

Recent studies have suggested indirect role of NOTCH pathway in glioma cell invasion. For instance, overexpression of NOTCH1 promotes the invasive capabilities of glioma cells [16], [37]. Therefore, we hypothesized that NOTCH3 may induce cell invasion in glioma based on similar oncogenic roles. We found that loss of NOTCH3 inhibits glioma cell migration and invasion and demonstrated that NOTCH3 regulates glioma cell invasion via interaction with the EGFR/PI3K/AKT signaling pathway because NOTCH3 Knockdown indeed reduced the expression of EGFR. These proteins are known key players in glioma cell invasion [16]. Together, these findings suggest that NOTCH3 is highly likely to induce glioma cell invasion and proliferation via at least the activation of CCND1, cMYC and EGFR gene expression.

Lastly, we reported that NOTCH3 knockdown resulted in cellular apoptosis shown by marked DNA fragmentation. We were interested to identify possible mechanisms for induction of such apoptosis following loss of NOTCH3 and found that NOTCH3 knockdown resulted in downregulation of EGFR which has also anti-apoptotic properties. In fact, previous studies have reported direct regulation of EGFR by NOTCH pathway [38], [39]. For instance, NICD1/CSL transcription complex has been reported to induce EGFR activity and this activation is abolished by NOTCH1 knockdown in other cancers [38]. Furthermore EGFR has been reported to mediate NOTCH-dependent regulation of the anti-apoptotic Mcl-1 protein at both transcriptional and post-translational level [39], [40]. Together, these results strongly indicate the anti-apoptotic effect of NOTCH3 in glioma may occur at least via modulation of EGFR. However, additional experiments are needed to assess the exact mechanism.

## Conclusion

Our findings elucidate, for the first time, the ability of NOTCH3 to alter aggressive behavior of glioma cells such as proliferation and invasion. This modulatory effect of NOTCH3 can be predominantly attributed to NOTCH target genes as well as its potential cooperation with major aberrant signaling pathways in malignant glioma such as EGFR, C-MYC and CCND1, which are all often altered in glioma. In addition, multiple NOTCH3 gene copy number as well as high NOTCH3 mRNA and protein levels in aggressive high grade astrocytic glioma may be explained by our findings about the prognostic value and the oncogenic role of NOTCH3.

NOTCH3 over-activation which subsequently results from the Chromosome 19 gain signature might be considered as a potential predictive factor. Therefore, NOTCH inhibition can be foreseen as a promising strategy in patients with chromosome 19/NOTCH3 amplifications. Hence, we hypothesize that targeting NOTCH3 with specific inhibitors such as γ-secretase inhibitors or with an anti-NOTCH3 antibody may improve therapeutic outcomes specifically in patients with this genetic signature. Our data suggest that such information, after being validated in pre-clinical and clinical models, might be used as prognostic and predictive marker for development of patient-specific approaches to individualize treatments.

## Supporting Information

Figure S1 **Colony formation of U87-MG cells.** Soft agar assay was used to assess anchorage-independent growth following shRNA treatment. Colonies were stained with crystal violet and visualized by light microscopy and counted from 10 independent fields.
(TIF)

Figure S2 **NOTCH3 promotes both migratory and invasive abilities of glioma cells.** **(A)** Time course of U87-MG cell migration following NOTCH3 knockdown. Confluent monolayers of U87-MG cells were scratched using a pipette tip. Wound images were captured with a digital camera attached to the microscope at 0, 12 & 24 hours. The dashed lines indicate the width of the wound. **(B)** Effect of NOTCH3 knockdown on the invasion of U87-MG cells. Invasion of the U87-MG cells was determined by measuring the ability of cells to pass through the matrigel coated membrane.
(TIF)
