# Supplementary figures and images for: NOTCH3 Is a Prognostic Factor That Promotes Glioma Cell Proliferation, Migration and Invasion via Activation of CCND1 and EGFR
Source: PLoS One. 2013 Oct 15;8(10):e77299. doi: 10.1371/journal.pone.0077299 (PMC3797092; doi:10.1371/journal.pone.0077299)

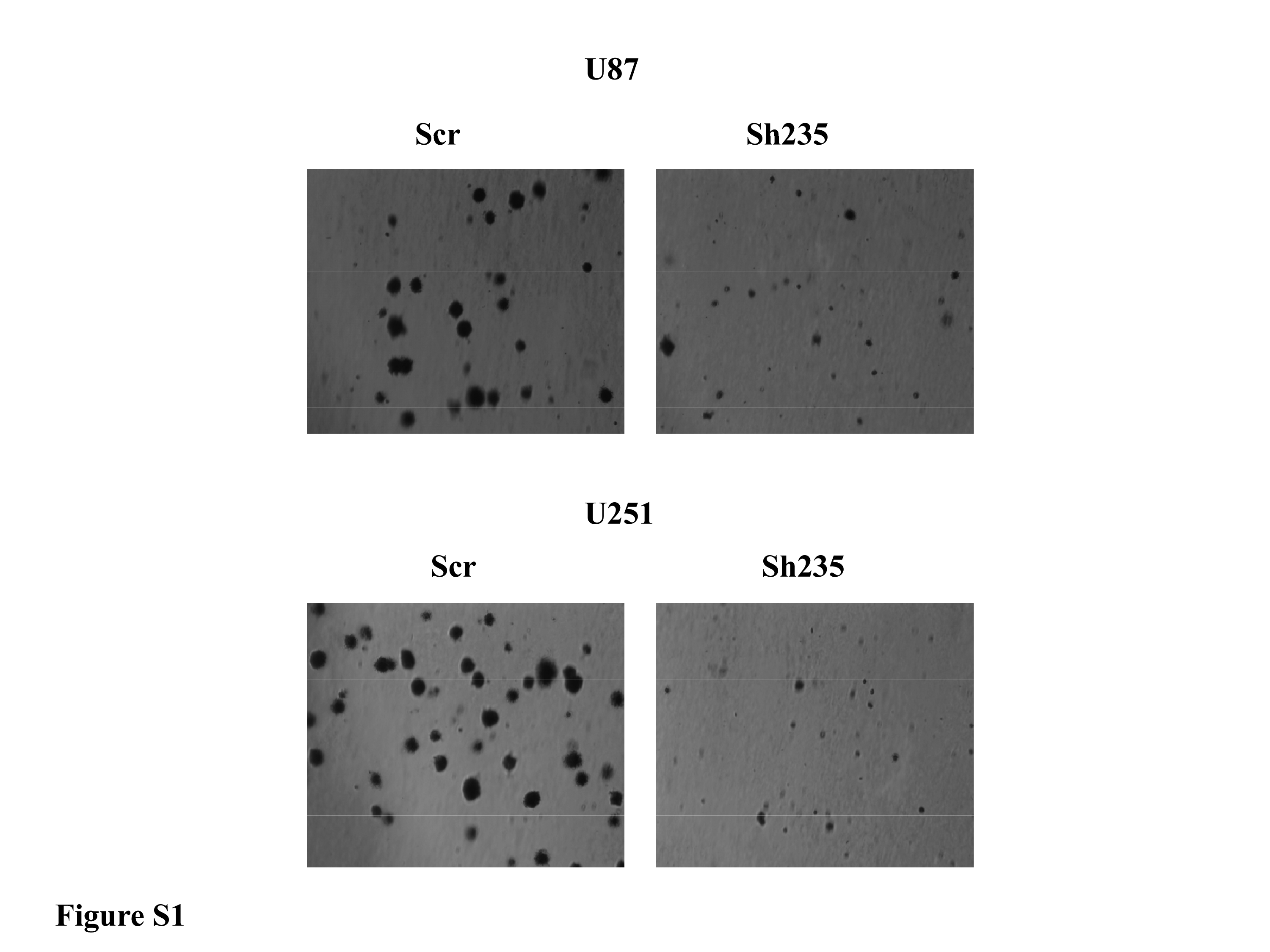

Supplement: Figure S1 — Colony formation of U87-MG cells. Soft agar assay was used to assess anchorage-independent growth following shRNA treatment. Colonies were stained with crystal violet and visualized by light microscopy and counted from 10 independent fields. (TIF) [file pone.0077299.s001.tif]

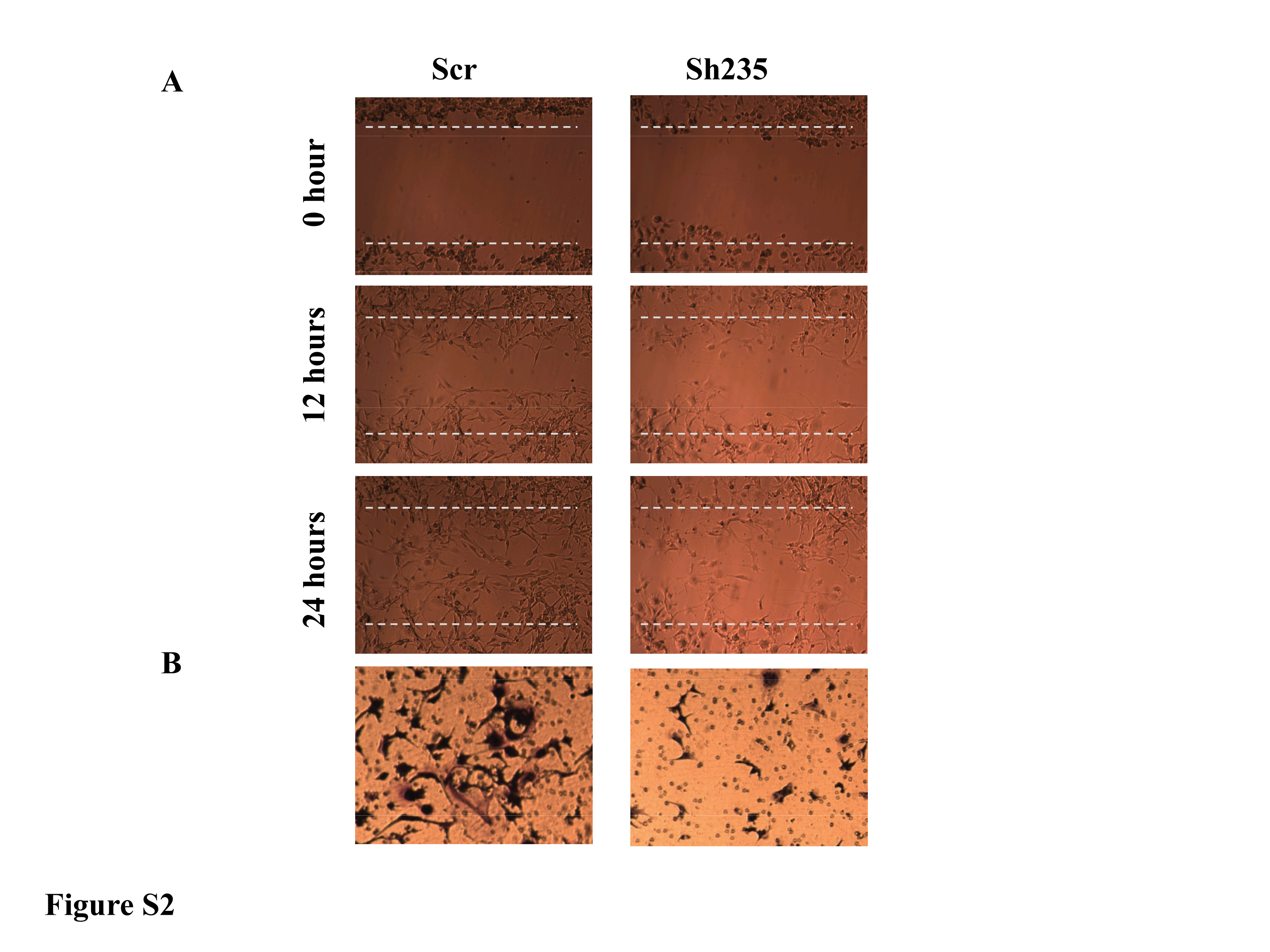

Supplement: Figure S2 — NOTCH3 promotes both migratory and invasive abilities of glioma cells. (A) Time course of U87-MG cell migration following NOTCH3 knockdown. Confluent monolayers of U87-MG cells were scratched using a pipette tip. Wound images were captured with a digital camera attached to the microscope at 0, 12 & 24 hours. The dashed lines indicate the width of the wound. (B) Effect of NOTCH3 knockdown on the invasion of U87-MG cells. Invasion of the U87-MG cells was determined by measuring the ability of cells to pass through the matrigel coated membrane. (TIF) [file pone.0077299.s002.tif]
